# 6-Azido-6-de­oxy-α-l-galactose (6-azido-l-fucose) monohydrate

**DOI:** 10.1107/S1600536808022563

**Published:** 2008-07-23

**Authors:** K. Victoria Booth, Sarah F. Jenkinson, Devendar Rao, Tsuyosi Simonisi, George W. J. Fleet, Ken Izumori, David J. Watkin

**Affiliations:** a Department of Organic Chemistry, Chemical Research Laboratory, University of Oxford, Mansfield Road, Oxford OX1 3TA, England; b Rare Sugar Research Centre, Kagawa University, 2393 Miki-cho, Kita-gun, Kagawa 761-0795, Japan; c Department of Chemical Crystallography, Chemical Research Laboratory, University of Oxford, Mansfield Road, Oxford OX1 3TA, England

## Abstract

Although 6-azido-6-de­oxy-l-galactose in aqueous solution is in equilibrium between the open-chain, furan­ose and pyran­ose forms, it crystallizes solely as 6-azido-6-de­oxy-α-l-galactopyran­ose monohydrate, C_6_H_11_N_3_O_5_·H_2_O, with the six-membered ring adopting a chair conformation. The structure exists as hydrogen-bonded chains, with each mol­ecule acting as a donor and acceptor of five hydrogen bonds. There are no unusual crystal packing features and the absolute configuration was determined from the use of 1-azido-1-de­oxy-d-galactitol as the starting material.

## Related literature

For related literature see: Beadle *et al.* (1992 ▶); Izumori (2002 ▶, 2006 ▶); Granstrom *et al.* (2004 ▶); Sun *et al.* (2007 ▶); Levin (2002 ▶); Skytte (2002 ▶); Nakajima *et al.* (2004 ▶); Sui *et al.* (2005 ▶); Hossain *et al.* (2006 ▶); Kolb & Sharpless (2003 ▶); Chesterton *et al.* (2006 ▶); Görbitz (1999 ▶); Larson (1970 ▶); Prince (1982 ▶); Watkin (1994 ▶); Yoshihara *et al.* (2008 ▶).
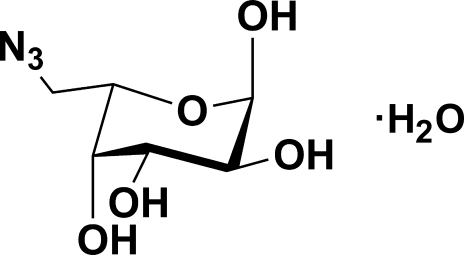


## Experimental

### 

#### Crystal data

C_6_H_11_N_3_O_5_·H_2_O
*M* *_r_* = 223.19
Orthorhombic, 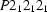

*a* = 5.9687 (3) Å
*b* = 7.7395 (4) Å
*c* = 20.9768 (11) Å
*V* = 969.02 (9) Å^3^
*Z* = 4
Mo *K*α radiation
μ = 0.14 mm^−1^
*T* = 150 K
0.50 × 0.05 × 0.05 mm

#### Data collection

Nonius KappaCCD diffractometer
Absorption correction: multi-scan *DENZO*/*SCALEPACK* (Otwinowski & Minor, 1997 ▶) *T*_min_ = 0.86, *T*_max_ = 0.99
7317 measured reflections
1296 independent reflections
792 reflections with *I* > 2σ(*I*)
*R*_int_ = 0.053

#### Refinement

*R*[*F* ^2^ > 2σ(*F* ^2^)] = 0.033
*wR*(*F* ^2^) = 0.073
*S* = 0.80
1095 reflections
136 parameters
H-atom parameters constrained
Δρ_max_ = 0.37 e Å^−3^
Δρ_min_ = −0.36 e Å^−3^

### 

Data collection: *COLLECT* (Nonius, 2001 ▶); cell refinement: *DENZO*/*SCALEPACK* (Otwinowski & Minor, 1997 ▶); data reduction: *DENZO*/*SCALEPACK*; program(s) used to solve structure: *SIR92* (Altomare *et al.*, 1994 ▶); program(s) used to refine structure: *CRYSTALS* (Betteridge *et al.*, 2003 ▶); molecular graphics: *CAMERON* (Watkin *et al.*, 1996 ▶); software used to prepare material for publication: *CRYSTALS*.

## Supplementary Material

Crystal structure: contains datablocks global, I. DOI: 10.1107/S1600536808022563/lh2654sup1.cif

Structure factors: contains datablocks I. DOI: 10.1107/S1600536808022563/lh2654Isup2.hkl

Additional supplementary materials:  crystallographic information; 3D view; checkCIF report

## Figures and Tables

**Table 1:** Hydrogen-bond geometry (Å, °)

*D*—H⋯*A*	*D*—H	H⋯*A*	*D*⋯*A*	*D*—H⋯*A*
O1—H11⋯O4^i^	0.81	1.96	2.760 (4)	169
O4—H41⋯O6^i^	0.83	1.83	2.648 (4)	171
O15—H151⋯O4^ii^	0.83	2.19	2.989 (4)	163
O8—H81⋯O15^iii^	0.83	1.90	2.732 (4)	177
O6—H62⋯O1^iv^	0.81	1.98	2.755 (4)	162

Symmetry codes: (i) 
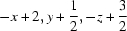
; (ii) 
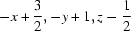
; (iii) 
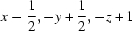
; (iv) 
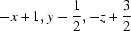
.

**Fig. 1.**
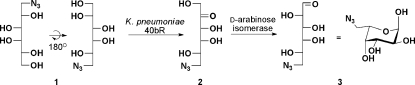
Synthetic scheme.

**Fig. 2.**
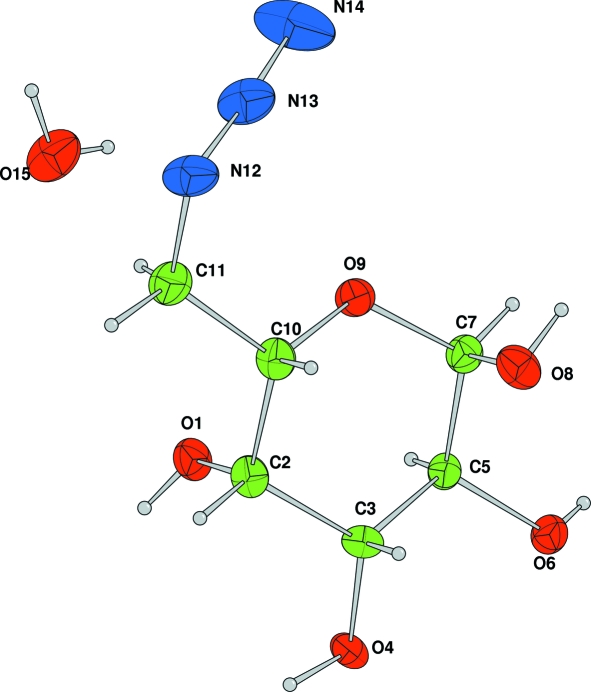
The title compound with displacement ellipsoids drawn at the 50% probability level. H atoms are shown as spheres of arbitary radius.

**Fig. 3.**
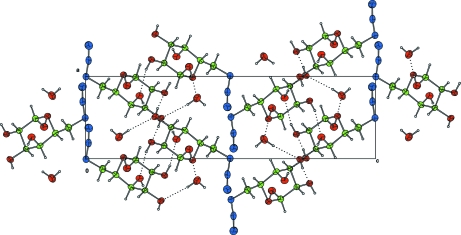
The packing diagram for the title compound projected along the b-axis.

## supplementary crystallographic information

### Comment

The range of rare sugars that are now readily available has increased in recent years due to both chemical (Beadle *et al.*, 1992) and biotechnological (Izumori, 2002,2006; Granstrom *et al.*, 2004) advances. Interest in rare sugars has been prompted by the search for low calorie alternative food stuffs (Sun *et al.*, 2007; Levin, 2002; Skytte, 2002) and also a potential range of other beneficial therapeutic properties (Nakajima *et al.*, 2004; Sui *et al.*, 2005; Hossain *et al.*, 2006).

The methodology developed by Izumori (2002,2006) for the interconversion of tetroses, pentoses and hexoses by enzymatic oxidation, inversion at C3 with a single epimerase, and reduction to the aldose has been seen to be generally applicable for the 1-deoxy ketohexoses (Yoshihara *et al.*, 2008). The viability of the methodology for the corresponding azido substituted systems was investigated with the synthesis 6-azido-6-deoxy-L-galactose 3 by microbial oxidation of 1-azido-1-deoxy-D-galactitol 1 with *K.Pneumoniae* 40bR followed by isomerization to the aldose 3 using D-arabinose isomerase (Fig. 1).

6-Azido-6-deoxy sugars have been little investigated and may have similar interesting properties. They are also of interest as Click Chemistry substrates, allowing a wide range of novel sugar substituted triazoles to be synthesized quickly, utilizing a few easy and reliable reactions. A click reaction should be wide in scope and easy to perform, use only readily available reagents, and be insensitive to oxygen and water. Reaction work-up and purification uses benign solvents and avoids chromatography. In many cases the reaction can be performed in, or on top of water; (Kolb and Sharpless, 2003) presenting an obvious environmental benefit to many existing precedures.

6-Azido-6-deoxy-L-galactose monohydrate crystallized solely in the α-pyranose form with the 6-membered ring adopting a chair conformation (Fig. 2). Each molecule acts as a donor and acceptor for 5 hydrogen bonds. A non standard hydrogen bond to the terminal azide nitrogen has been removed from the packing diagrams. The structure exists as discrete chains of molecules run ning parallel to the *a*-axis and exhibits no unusual crystal packing features. As is common with these materials, the azide group is non linear [N12—N13—N14 171.91° (6)] (Chesterton *et al.* 2006).

### Experimental

The title compound was crystallized from water: m.p. 345 - 348K; [α]_D_^21^-52.3 (*c*, 1.05 in H_2_O).

### Refinement

In the absence of significant anomalous scattering, Friedel pairs were merged. The relatively large ratio of minimum to maximum corrections applied in the multiscan process (1:1.15) reflect changes in the illuminated volume of the crystal. Changes in illuminated volume were kept to a minimum, and were taken into account (Görbitz, 1999) by the multi-scan inter-frame scaling (*DENZO*/*SCALEPACK*, Otwinowski & Minor, 1997).

The H atoms were all located in a difference map, but those attached to carbon atoms were repositioned geometrically. The H atoms were initially refined with soft restraints on the bond lengths and angles to regularize their geometry (C—H in the range 0.93–0.98, O—H = 0.82 Å) and *U*_iso_(H) (in the range 1.2–1.5 times *U*_eq_ of the parent atom), after which the positions were refined with riding constraints.

A few very weak reflections were ignored in the refinement, and was therefore carried out on only 1095 reflections, not the full 1296 originally collected.

### Crystal data

**Table:**

C_6_H_11_N_3_O_5_·H_2_O	*F*_000_ = 472
*M**_r_* = 223.19	*D*_x_ = 1.530 Mg m^−^^3^
Orthorhombic, *P*2_1_2_1_2_1_	Mo *K*α radiation λ = 0.71073 Å
Hall symbol: P 2ac 2ab	Cell parameters from 2018 reflections
*a* = 5.9687 (3) Å	θ = 5–27º
*b* = 7.7395 (4) Å	µ = 0.14 mm^−^^1^
*c* = 20.9768 (11) Å	*T* = 150 K
*V* = 969.02 (9) Å^3^	Plate, colourless
*Z* = 4	0.50 × 0.05 × 0.05 mm

### Data collection

**Table:**

Nonius KappaCCD diffractometer	792 reflections with *I* > 2σ(*I*)
Monochromator: graphite	*R*_int_ = 0.053
*T* = 150 K	θ_max_ = 27.4º
ω scans	θ_min_ = 5.2º
Absorption correction: multi-scanDENZO/SCALEPACK (Otwinowski & Minor, 1997)	*h* = −7→7
*T*_min_ = 0.86, *T*_max_ = 0.99	*k* = −9→10
7317 measured reflections	*l* = −26→27
1296 independent reflections	

### Refinement

**Table:**

Refinement on *F*^2^	Hydrogen site location: inferred from neighbouring sites
Least-squares matrix: full	H-atom parameters constrained
*R*[*F*^2^ > 2σ(*F*^2^)] = 0.033	*w* = 1/[σ^2^(*F*^2^)]
*wR*(*F*^2^) = 0.073	(Δ/σ)_max_ = 0.0003
*S* = 0.80	Δρ_max_ = 0.37 e Å^−^^3^
1095 reflections	Δρ_min_ = −0.36 e Å^−^^3^
136 parameters	Extinction correction: None
Primary atom site location: structure-invariant direct methods	

### Fractional atomic coordinates and isotropic or equivalent isotropic displacement parameters (Å^2^)

**Table:**

	*x*	*y*	*z*	*U*_iso_*/*U*_eq_	
O1	0.7366 (3)	0.6574 (2)	0.68646 (7)	0.0240	
C2	0.8440 (4)	0.5120 (3)	0.65721 (11)	0.0207	
C3	0.8824 (4)	0.3657 (4)	0.70492 (11)	0.0198	
O4	1.0088 (3)	0.4176 (3)	0.76006 (7)	0.0239	
C5	0.6592 (4)	0.2991 (4)	0.72879 (11)	0.0185	
O6	0.7020 (3)	0.1614 (2)	0.77263 (7)	0.0218	
C7	0.5141 (4)	0.2396 (3)	0.67315 (11)	0.0203	
O8	0.6145 (3)	0.0996 (3)	0.64297 (8)	0.0276	
O9	0.4833 (3)	0.3840 (2)	0.63098 (7)	0.0228	
C10	0.6911 (4)	0.4467 (4)	0.60441 (11)	0.0223	
C11	0.6234 (5)	0.5891 (4)	0.55904 (11)	0.0286	
N12	0.5055 (4)	0.5214 (3)	0.50147 (10)	0.0336	
N13	0.3088 (4)	0.4780 (4)	0.51035 (10)	0.0347	
N14	0.1278 (4)	0.4350 (5)	0.51097 (11)	0.0585	
O15	0.7358 (3)	0.5841 (3)	0.38440 (7)	0.0372	
H21	0.9866	0.5468	0.6385	0.0251*	
H31	0.9606	0.2684	0.6830	0.0246*	
H51	0.5793	0.3928	0.7511	0.0236*	
H71	0.3616	0.2056	0.6878	0.0253*	
H101	0.7643	0.3512	0.5817	0.0281*	
H111	0.7596	0.6432	0.5432	0.0344*	
H112	0.5329	0.6774	0.5803	0.0343*	
H152	0.6532	0.5377	0.4105	0.0561*	
H11	0.8239	0.7312	0.6983	0.0373*	
H41	1.1103	0.4866	0.7514	0.0381*	
H151	0.6582	0.6044	0.3527	0.0563*	
H81	0.5011	0.0423	0.6335	0.0441*	
H62	0.5844	0.1468	0.7909	0.0334*	

### Atomic displacement parameters (Å^2^)

**Table:**

	*U*^11^	*U*^22^	*U*^33^	*U*^12^	*U*^13^	*U*^23^
O1	0.0231 (9)	0.0184 (10)	0.0306 (9)	0.0001 (9)	0.0005 (9)	−0.0023 (9)
C2	0.0192 (13)	0.0202 (16)	0.0226 (12)	0.0007 (13)	0.0055 (12)	−0.0010 (13)
C3	0.0164 (12)	0.0236 (18)	0.0193 (12)	0.0008 (13)	−0.0022 (11)	−0.0033 (13)
O4	0.0215 (9)	0.0256 (11)	0.0247 (9)	−0.0089 (9)	−0.0044 (8)	0.0023 (9)
C5	0.0192 (13)	0.0169 (16)	0.0193 (12)	−0.0005 (12)	0.0002 (11)	0.0020 (13)
O6	0.0193 (9)	0.0225 (11)	0.0236 (8)	0.0003 (9)	0.0017 (8)	0.0046 (10)
C7	0.0217 (13)	0.0184 (14)	0.0207 (13)	0.0012 (14)	−0.0002 (13)	−0.0003 (13)
O8	0.0269 (10)	0.0248 (11)	0.0310 (9)	−0.0028 (10)	−0.0001 (9)	−0.0065 (10)
O9	0.0194 (9)	0.0267 (11)	0.0223 (9)	−0.0011 (9)	0.0005 (8)	0.0043 (9)
C10	0.0218 (14)	0.0238 (16)	0.0213 (12)	0.0001 (13)	0.0043 (12)	0.0004 (13)
C11	0.0293 (15)	0.0334 (17)	0.0232 (13)	−0.0028 (16)	−0.0001 (12)	0.0066 (15)
N12	0.0253 (12)	0.0542 (19)	0.0213 (11)	−0.0009 (13)	0.0003 (11)	0.0049 (13)
N13	0.0364 (15)	0.0501 (19)	0.0174 (13)	0.0037 (14)	0.0003 (11)	0.0006 (13)
N14	0.0350 (16)	0.107 (3)	0.0336 (15)	−0.0156 (19)	0.0022 (14)	−0.0111 (19)
O15	0.0357 (10)	0.0476 (13)	0.0283 (9)	0.0078 (12)	0.0071 (9)	0.0092 (11)

### Geometric parameters (Å, °)

**Table:**

O1—C2	1.433 (3)	C7—O9	1.437 (3)
O1—H11	0.812	C7—H71	0.997
C2—C3	1.529 (3)	O8—H81	0.834
C2—C10	1.522 (3)	O9—C10	1.444 (3)
C2—H21	0.975	C10—C11	1.511 (4)
C3—O4	1.438 (3)	C10—H101	0.982
C3—C5	1.514 (3)	C11—N12	1.493 (3)
C3—H31	0.999	C11—H111	0.973
O4—H41	0.828	C11—H112	0.978
C5—O6	1.431 (3)	N12—N13	1.235 (3)
C5—C7	1.524 (3)	N13—N14	1.130 (3)
C5—H51	0.986	O15—H152	0.820
O6—H62	0.807	O15—H151	0.825
C7—O8	1.391 (3)		
			
C2—O1—H11	113.3	C5—C7—O9	108.0 (2)
O1—C2—C3	111.62 (19)	O8—C7—O9	112.39 (18)
O1—C2—C10	107.7 (2)	C5—C7—H71	111.2
C3—C2—C10	108.7 (2)	O8—C7—H71	109.2
O1—C2—H21	110.3	O9—C7—H71	106.2
C3—C2—H21	109.7	C7—O8—H81	100.0
C10—C2—H21	108.8	C7—O9—C10	112.87 (18)
C2—C3—O4	113.5 (2)	C2—C10—O9	110.25 (19)
C2—C3—C5	109.7 (2)	C2—C10—C11	112.1 (2)
O4—C3—C5	106.90 (18)	O9—C10—C11	104.97 (19)
C2—C3—H31	109.1	C2—C10—H101	109.6
O4—C3—H31	109.6	O9—C10—H101	108.5
C5—C3—H31	107.9	C11—C10—H101	111.2
C3—O4—H41	112.7	C10—C11—N12	112.3 (3)
C3—C5—O6	108.02 (19)	C10—C11—H111	107.7
C3—C5—C7	110.46 (18)	N12—C11—H111	105.6
O6—C5—C7	111.6 (2)	C10—C11—H112	111.8
C3—C5—H51	109.4	N12—C11—H112	110.7
O6—C5—H51	109.2	H111—C11—H112	108.4
C7—C5—H51	108.1	C11—N12—N13	114.9 (2)
C5—O6—H62	104.7	N12—N13—N14	171.9 (3)
C5—C7—O8	109.8 (2)	H152—O15—H151	106.5

### Hydrogen-bond geometry (Å, °)

**Table:**

*D*—H···*A*	*D*—H	H···*A*	*D*···*A*	*D*—H···*A*
O15—H152···N12	0.82	2.11	2.856 (4)	152
O1—H11···O4^i^	0.81	1.96	2.760 (4)	169
O4—H41···O6^i^	0.83	1.83	2.648 (4)	171
O15—H151···O4^ii^	0.83	2.19	2.989 (4)	163
O8—H81···O15^iii^	0.83	1.90	2.732 (4)	177
O6—H62···O1^iv^	0.81	1.98	2.755 (4)	162

Symmetry codes: (i) −*x*+2, *y*+1/2, −*z*+3/2; (ii) −*x*+3/2, −*y*+1, *z*−1/2; (iii) *x*−1/2, −*y*+1/2, −*z*+1; (iv) −*x*+1, *y*−1/2, −*z*+3/2.

## References

[bb1] Altomare, A., Cascarano, G., Giacovazzo, C., Guagliardi, A., Burla, M. C., Polidori, G. & Camalli, M. (1994). *J. Appl. Cryst.* **27**, 435.

[bb2] Beadle, J. R., Saunders, J. P. & Wajda, T. J. (1992). US Patent 5 078 796.

[bb3] Betteridge, P. W., Carruthers, J. R., Cooper, R. I., Prout, K. & Watkin, D. J. (2003). *J. Appl. Cryst.* **36**, 1487.

[bb4] Chesterton, A. K. S., Jenkinson, S. F., Jones, N. A., Fleet, G. W. J. & Watkin, D. J. (2006). *Acta Cryst.* E**62**, o2983–o2985.

[bb5] Görbitz, C. H. (1999). *Acta Cryst.* B**55**, 1090–1098. 10.1107/s0108768199008721 10927450

[bb6] Granstrom, T. B., Takata, G., Tokuda, M. & Izumori, K. (2004). *J. Biosci. Bioeng.* **97**, 89–94. 10.1016/S1389-1723(04)70173-5 16233597

[bb7] Hossain, M. A., Wakabayashi, H., Izuishi, K., Okano, K., Yachida, S., Tokuda, M., Izumori, K. & Maeta, H. (2006). *J. Biosci. Bioeng.* **101**, 369–371. 10.1263/jbb.101.369 16716947

[bb8] Izumori, K. (2002). *Naturwissenschaften*, **89**, 120–124. 10.1007/s00114-002-0297-z 12046631

[bb9] Izumori, K. (2006). *J. Biotechnol.* **124**, 717–722. 10.1016/j.jbiotec.2006.04.016 16716430

[bb10] Kolb, H. C. & Sharpless, K. B. (2003). *Drug Discovery Today*, **8**, 1128–???. 10.1016/s1359-6446(03)02933-7 14678739

[bb11] Larson, A. C. (1970). *Crystallographic Computing*, edited by F. R. Ahmed, S. R. Hall & C. P. Huber, pp. 291–294. Copenhagen: Munksgaard.

[bb12] Levin, G. V. (2002). *J. Med. Food*, **5**, 23–36. 10.1089/109662002753723197 12511110

[bb13] Nakajima, Y., Gotanda, T., Uchimiya, H., Furukawa, T., Haraguchi, M., Ikeda, R., Sumizawa, T., Yoshida, H. & Akiyama, S. (2004). *Cancer Res.* **64**, 1794–1801. 10.1158/0008-5472.can-03-2597 14996742

[bb14] Nonius (2001). *COLLECT* Nonius BV, Delft, The Netherlands.

[bb15] Otwinowski, Z. & Minor, W. (1997). *Methods in Enzymology*, Vol. 276, *Macromolecular Crystallography*, Part A, edited by C. W. Carter Jr & R. M. Sweet, pp. 307–326. New York: Academic Press.

[bb16] Prince, E. (1982). *Mathematical Techniques in Crystallography and Materials Science* New York: Springer-Verlag.

[bb17] Skytte, U. P. (2002). *Cereal Foods World*, **47**, 224–224.

[bb18] Sui, L., Dong, Y. Y., Watanabe, Y., Yamaguchi, F., Hatano, N., Tsukamoto, I., Izumori, K. & Tokuda, M. (2005). *Int. J. Oncol* **27**, 907–912. 16142305

[bb19] Sun, Y. X., Hayakawa, S., Ogawa, M. & Izumori, K. (2007). *Food. Contr.* **18**, 220–227.

[bb20] Watkin, D. (1994). *Acta Cryst.* A**50**, 411–437.

[bb21] Watkin, D. J., Prout, C. K. & Pearce, L. J. (1996). *CAMERON* Chemical Crystallography Laboratory, Oxford, UK.

[bb22] Yoshihara, A., Haraguchi, S., Gullapalli, P., Rao, D., Morimoto, K., Takata, G., Jones, N., Jenkinson, S. F., Wormald, M. R., Dwek, R. A., Fleet, G. W. J. & Izumori, K. (2008). *Tetrahedron Asymmetry*, **19**, 739–745.

